# Supplementary material for: Epigenetic regulation of DPP4 receptor expression by NONO enables replication of MERS-CoV
Source: bioRxiv. 2026 Sep 23:2026.09.21.753343. Preprint. [Version 1] doi: 10.64898/2026.09.21.753343 (PMC13622116; doi:10.64898/2026.09.21.753343)

bioRxiv preprint doi: https://doi.org/10.64898/2026.09.21.753343; this version posted September 23, 2026. The copyright holder for this preprint (which was not certified by peer review) is the author/funder. This article is a US Government work. It is not subject to copyright under 17 USC 105 and is also made available for use under a CC0 license.

Figure S1: NONO is an essential host factor for MERS-CoV replication:
(A) Infectious viral titers from hACE2/hTMPRSS2 expressing A549 cells treated with siNTC or siNONO infected with SARS-CoV-2 (MOI=0.01) (plaque assay). (B-C) GSEA terms and pathways of down and upregulated transcripts from WT or *NONO* KO HAP1 samples. Numbers adjacent to bubbles represent gene counts. (D) Gating strategy to determine DPP4 surface expression from WT or *NONO* KO HAP1 cells (flow cytometry). Data are expressed as means (n=3) ± SD, **p < 0.01, (two-way ANOVA with Sidak's multiple comparisons) and are representative of 2-3 independent experiments.

Figure S2: NONO deletion disrupts DPP4 transcription and MERS-CoV entry:
(A) Infectious viral titers from WT, *NONO* KO, and *DPP4* KO HAP1 cells infected with MERS-CoV (MOI=0.01) (plaque assay). (B) Protein levels for DPP4 and NONO from WT, *NONO* KO, and *DPP4* KO HAP1 cells (immunoblot). (C) DPP4 surface expression from DPP4 transduced or untransduced WT or *NONO* KO HAP1 cells (flow cytometry). (D) WT or *NONO* KO HAP1 cells infected with MERS-CoV (MOI=0.01) zoom in of 48-72 hpi infectious viral titers from (Figure 3H) (plaque assay). (E-F) WT HAP1 cells infected with MERS-CoV (MOI=0.01) for 24 hours. (E) Subcellular fractionation (immunoblot). (F) Expression of NONO-bound MERS-CoV vRNA from endogenous NONO RNA immunoprecipitation (qRT-PCR). Data are expressed as means (n=3) ± SD, *p < 0.05, **p < 0.01, ****p < 0.0001 (two-way ANOVA with Dunnett's or Tukey's multiple comparisons or Welch's t-test) and are representative of 2-3 independent experiments.

Figure S3: NONO couples chromatin remodeling to expression of the MERS-CoV entry receptor:
(A-B) Endogenous NONO RNA immunoprecipitation from WT HAP1 cells. (A) NONO immunoprecipitation efficiency (immunoblot). (B) Expression of NONO-bound *DPP4* (qRT-PCR). (C) ChIP-qPCR showing H3K27me3 and H3K4me3 histone modifications at *RPL30* and *α-Satellite* from WT or *NONO* KO HAP1 cells. Data are expressed as means (n=3) ± SD, ***p < 0.001, (Welch's t-test) and are representative of 2-3 independent experiments.

bioRxiv preprint doi: https://doi.org/10.64898/2026.09.21.753343; this version posted September 23, 2026. The copyright holder for this preprint (which was not certified by peer review) is the author/funder. This article is a US Government work. It is not subject to copyright under 17 USC 105 and is also made available for use under a CC0 license.

Table S1: Primer sequences

REAGENT or RESOURCE	FORWARD	REVERSE
Oligonucleotides for qRT-PCR		
18S rRNA	5’-GTAACCCGTTGAACCCCATT-3’	5’-CCATCCAATCGGTAGTAGCG-3’
IFNB1	5’-TCTGGCACAACAGGTAGTAGGC-3’	5’-GAGAAGCACAACAGGAGAGCAA-3’
IFNL2	5’-TCGCTTCTGCTGAAGGACTGCA-3’	5’-CCTCCAGAACCTTCAGCGTCAG-3’
IFIT1 [47]	5’-GCCTTGCTGAAGTGTGGAGGAA-3’	5’-ATCCAGGCGATAGGCAGAGATC-3’
NONO	5’-CATCAAGGAGGCTCGTGAGAAG-3’	5’-TGGTTGTGCAGCTCTTCCATCC-3’
MERS-CoV ORF1b [48]	5’-TTCGATGTTGAGGGTGCTCAT-3’	5’-TCACACCAGTTGAAAATCCTAATTG-3’
MERS-CoV upE [48]	5’-GCAACGCGCGATTCAGTT-3’	5’-GCCTCTACACGGGACCCATA-3’
MERS-CoV M mRNA [49,50]	5’-CTATCTCACTTCCCCTCGTTCTC-3’	5’-GGAAGATGGCCATAGGAGCC-3’
ACE2	5’-TCCATTGGTCTTCTGTCACCCG-3’	5’-AGACCATCCACCTCCACTTCTC-3’
TMPRSS2	5’-CCTCTAACTGGTGTGATGGCGT-3’	5’-TGCCAGGACTTCCTCTGAGATG-3’
DPP4	5’-AAAGGCACCTGGGAAGTCATCG-3’	5’-CAGCTCACAACTGAGGCATGTC-3’
DPP4 pre-mRNA	5’-ACAGCTGACAGTCGCAAAAC-3’	5’-TCAGTGCCATAAAAGCCCACA-3’
Oligonucleotides for ChIP-PCR		

bioRxiv preprint doi: https://doi.org/10.64898/2026.09.21.753343; this version posted September 23, 2026. The copyright holder for this preprint (which was not certified by peer review) is the author/funder. This article is a US Government work. It is not subject to copyright under 17 USC 105 and is also made available for use under a CC0 license.

DPP4 gene body	5'-CATGGCCAACAGTTCAGGC-3'	5'-GTGGGAAACAGGCAAAAGAGTT-3'
RPL30	Cell Signaling Technology Cat# 7014	
α-Satellite	Cell Signaling Technology Cat# 4486	

bioRxiv preprint doi: https://doi.org/10.64898/2026.09.21.753343; this version posted September 23, 2026. The copyright holder for this preprint (which was not certified by peer review) is the author/funder. This article is a US Government work. It is not subject to copyright under 17 USC 105 and is also made available for use under a CC0 license.

FIG S1

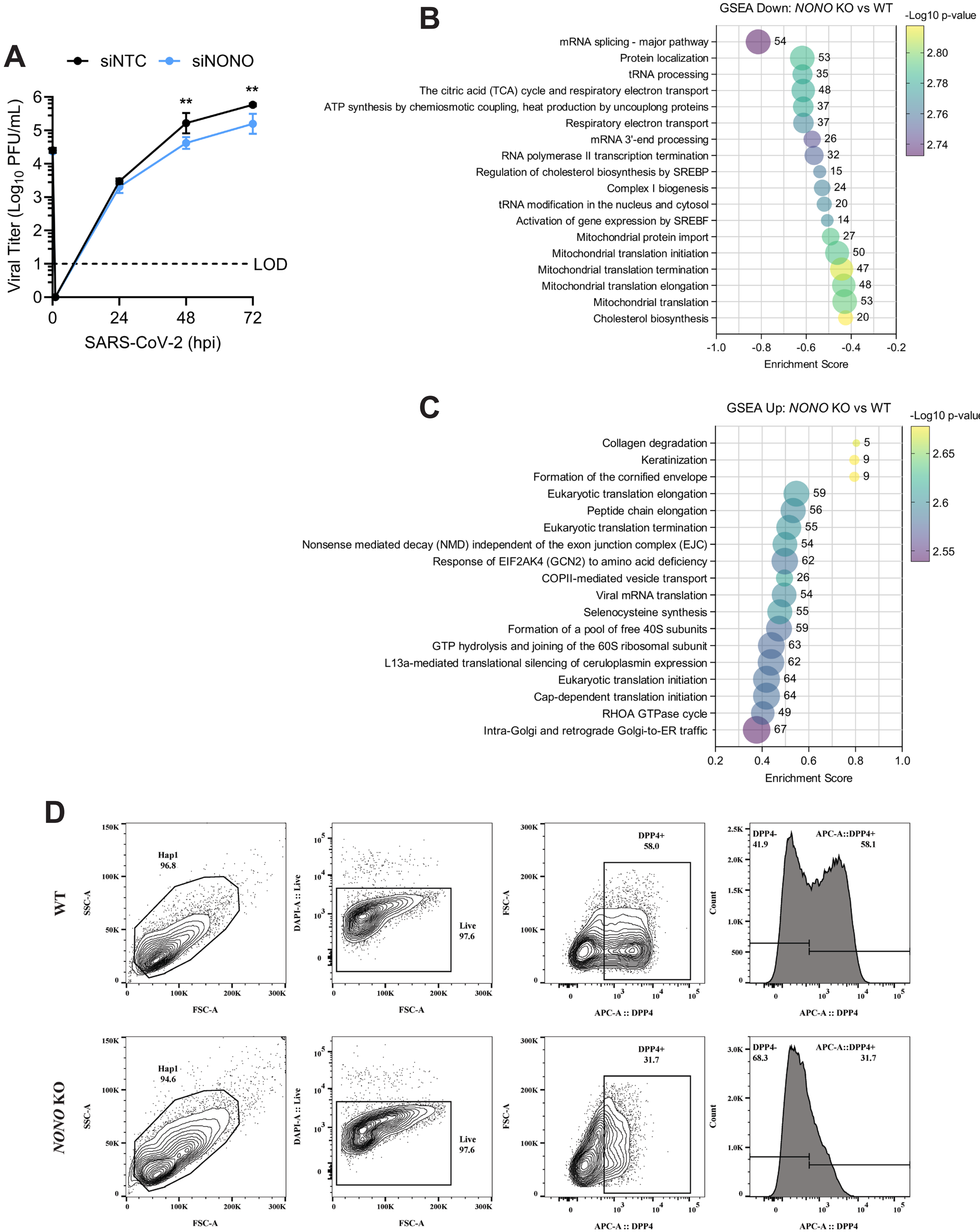

bioRxiv preprint doi: https://doi.org/10.64898/2026.09.21.753343; this version posted September 23, 2026. The copyright holder for this preprint (which was not certified by peer review) is the author/funder. This article is a US Government work. It is not subject to copyright under 17 USC 105 and is also made available for use under a CC0 license.

FIG S2

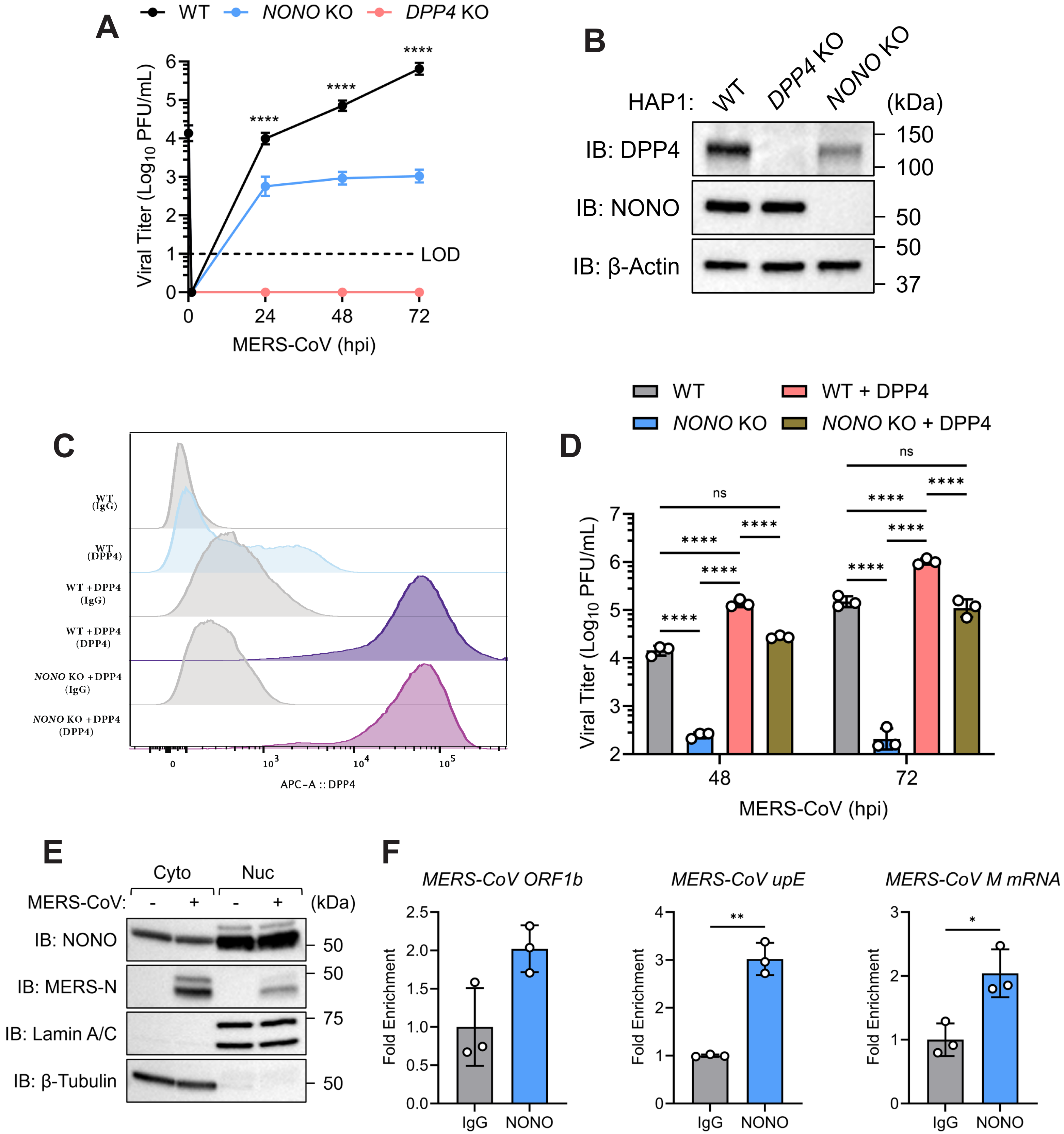

bioRxiv preprint doi: https://doi.org/10.64898/2026.09.21.753343; this version posted September 23, 2026. The copyright holder for this preprint (which was not certified by peer review) is the author/funder. This article is a US Government work. It is not subject to copyright under 17 USC 105 and is also made available for use under a CC0 license.

FIG S3

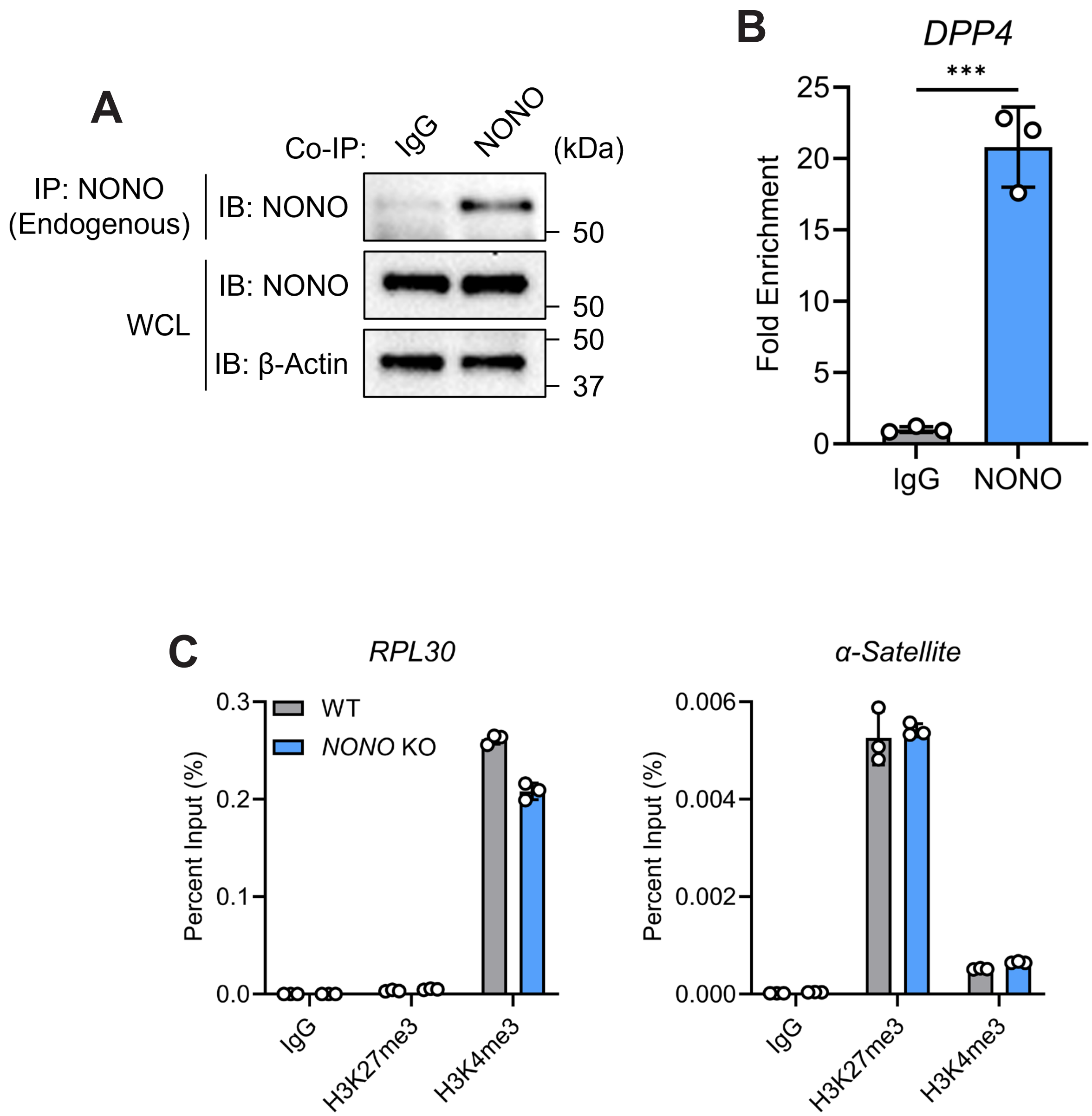

Supplement: Supplementary file 1 [file NIHPP2026.09.21.753343v1-supplement-1.pdf]
